# Oxidative downmodulation of T cell-mediated cytotoxicity

**DOI:** 10.1038/cddis.2016.273

**Published:** 2016-09-22

**Authors:** Guido H Wabnitz, Yvonne Samstag

**Affiliations:** 1 Institute of Immunology, Section Molecular Immunology, Ruprecht-Karls-University, Heidelberg, Germany

The cellular microenvironment influences the outcome of immune responses. Important factors of the microenvironment are reactive oxygen species that are produced by, for example, tumor cells or infiltrating myeloid cells. A misbalance in the production of reactive oxygen species (oxidative stress) is a hallmark of the pathogenesis of a variety of disorders, for example, chronic infection, tissue inflammation, degenerative diseases and cancer.^1^ Upon exposure to oxidative stress, T cells become insensitive with respect to receptor stimulation that dampens an immune response. In this regard, we have shown that pro-oxidative conditions induce hyporesponsiveness of primary human T cells via oxidation of the actin-binding protein cofilin^2, 3^ (reviewed in Samstag *et al.*^4^). Although T cell hyporesponsiveness is detrimental in tumor settings, it can be beneficial in certain immune-mediated inflammatory diseases (IMIDs) or to prevent graft loss after organ transplantation. Thus, a pharmacological modulation of the redox microenvironment may be efficient to control progression of IMIDs or to avoid graft rejection.

WF10 is a pro-oxidative drug that generates active chlorite species upon interaction with heme iron proteins.^5^ Moreover, we showed that it induced reactive oxygen species in human cytotoxic T-cells (CTLs).^6^ Importantly, WF10 was designed for intravenous injections,^7^ allowing clinical use of this compound. Indeed, WF10 entered clinical practise for treatment of chronic inflammatory disorders such as proctitis, cystitis, mucositis^8^ or diabetic foot ulcer (DFU).^9^ In our current work, we found that WF10 inhibits CTL-mediated target cell killing in a dose-dependent manner,^6^ providing a potential explanation of why graft survival in a concordant xenograft model was significantly prolonged in the presence of WF10,^10^ and why WF10 improves the clinical outcome of DFU.^9^

During target cell killing, CTLs firmly attach to target cells and form cytolytic immune synapses (Figure 1a). Lytic granules are released into the respective synaptic cleft that finally leads to the onset of apoptosis in the target cells. To efficiently clear all harmful cells, each CTL has to kill several targets. For such a serial killing CTLs perform rounds of target cell attachment, killing and detachment (Figure 1a, upper row). Unexpectedly, WF10 did not interfere with molecular mechanisms involved in degranulation of CTLs. Instead, we found that WF10 interfered with detachment of CTLs from target cells (Figure 1a, lower panel).^6^ This increased dwell time led to a significant slowing down of serial killing and an increased survival of target cells.

Serial killing requires sequential adhesion and deadhesion of T cells to target cells. A major adhesion molecule of T cells is LFA-1. Adhesive properties of LFA-1 can be regulated by two mechanisms, affinity and avidity. Whereas affinity upregulation increases the adhesion properties of single receptors, avidity is increased by formation of LFA-1 clusters.^11^ Avidity is upregulated in the cytolytic immune synapse that is important for target cell killing (Figure 1b, upper row). In order to release the dying target cell, LFA-1 avidity is downregulated, enabling CTLs to engage other target cells and to perform serial killing. Thus, an LFA-1 avidity up- and downregulating circle enables serial killing by CTLs. WF10 acts on this molecular switch by prolonging LFA-1 avidity on CTLs (Figure 1b, lower panel).^6^ L-plastin (LPL), an actin-bundling protein, is one regulator of LFA-1 avidity in the cytoplasm (Figure 1b, upper row) that connects LFA-1 to the actin cytoskeleton.^12^ The activity of L-plastin in human T cells increases by phosphorylation on serine-5.^13^ Such a phosphorylation is only transient in CTLs that are attached to their target cells.^6^ Thus, the initial phosphorylation of L-plastin enables LFA-1-dependent CTL adhesion to the target cell and, thereby, target cell killing. The subsequent L-plastin dephosphorylation allows the detachment of the CTL from the dying target cell. The reversible phosphorylation of L-plastin and the resulting LFA-1 avididiy regulation can therefore be considered as an internal impulse generator for serial killing by CTLs. WF10 provokes a constant phosphorylation of L-plastin and, consequently, an ongoing increase in LFA-1 avidity leading to an inhibition of serial killing. It is currently not known whether such a continuous L-plastin phosphorylation in the presence of WF10 is due to an increased kinase activity, decreased phosphatase activity or a structural change of L-plastin. The functional relevance of L-plastin for the inhibitory effect of WF10 was, however, certified by the finding that WF10 lost its influence on CTL-mediated killing in L-plastin knockdown CTLs.

CTLs identify and eliminate infected or transformed cells and are therefore important for the healthiness of an individual. The other side of the coin is that CTLs have the potential to kill healthy cells, thereby exerting detrimental functions, for example in certain IMIDs, such as type IV hypersensitivity reaction. Therefore, CTLs need to be tightly regulated and inhibition of CTL-mediated killing has an enormous potential for therapeutic immunosuppression. Most immunosuppressive drugs develop their effects by interfering with gene transcription and/or cell proliferation during the proximal phase of T cell activation. There are only few reports describing effects of immunosuppressive drugs on the distal phase of T cell-mediated immune responses and the cytolytic immune synapse. Particularly, only limited inhibitory effects on CTL-mediated target cell killing were reported for some immunosuppressants.^6, 14, 15^ Given that (1) cytotoxic T cells are important during transplant rejection or certain IMIDs and (2) the proximal T cell activation has most often already occurred at the time point of the diagnosis of IMIDs, a more effective suppression of T cell effector functions would be desirable. The pro-oxidative drug WF10 not only interferes significantly with serial killing, but also synergizes with the calcineurin inhibitors CsA and FK506, enabling or enforcing the inhibition of CTL-mediated target cell killing.^6^ This is of special importance as CsA alone showed no effect on CTL-mediated killing. Therefore, WF10 opens the possibility of providing a non-overlapping but synergizing therapy to treat IMID patients or avoiding graft loss after organ transplantation.

## Figures and Tables

**Figure 1:**
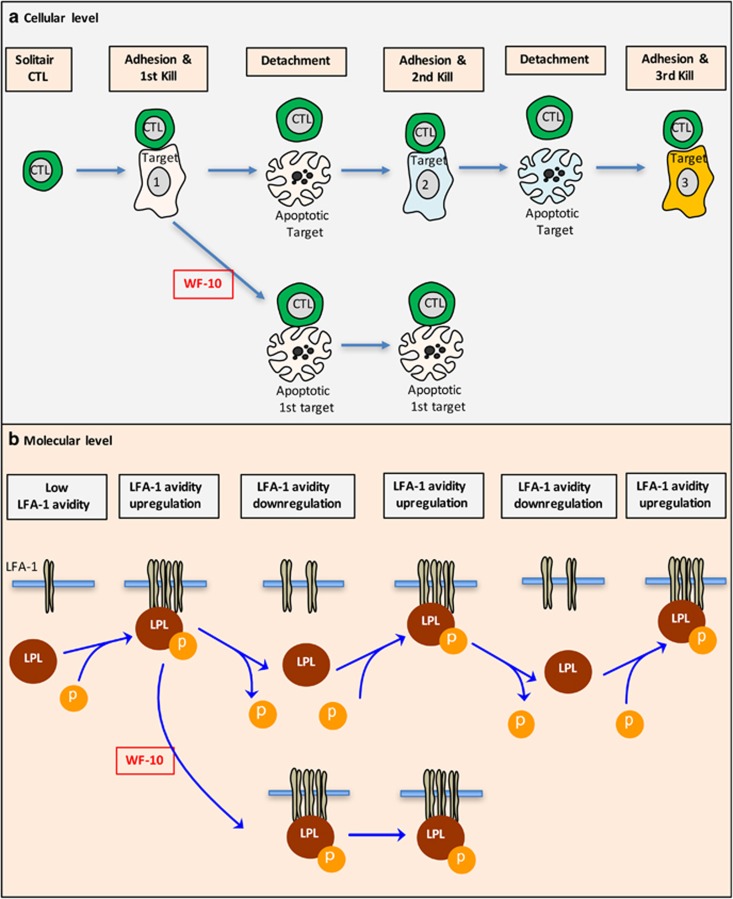
Cellular and molecular regulation of serial killing and its inhibition by WF10. (**a**) Cellular level. Cytotoxic T cells (CTLs) migrate as solitaire cells during the immune surveillance into inflammed tissues in order to find target cells. After encountering a target cells, CTLs firmly adhere to these cells and induce their apoptosis (first kill, upper row). To kill a second target cell, CTLs need to detach from the dying target cell and attach to a second target cell, perform the second kill and so on. One CTL can kill more than 6 target cells in a row. WF10 interferes with detachment of the CTL from their initial target cell (lower row). This leads to a strong decrease in the killing frequency of CTLs. (**b**) Molecular level. The attachment/detachment cycle during serial killing is dependent on an LFA-1 avidity up- and down regulation circle (upper row). The molecular motor regulating the LFA-1 avidity is the actin bundling protein L-plastin (LPL). L-plastin is transiently phosphorylated upon target cell encounter. Only the dephosphorylation of L-plastin enables the downregulation of LFA-1 avidity and the detachment of CTLs from the target cell. WF10 shifts the balance toward phosphorylated L-plastin by an as yet unknown mechanism and, thereby, prevents serial killing (lower row)
